# Pol α-primase dependent nuclear localization of the mammalian CST complex

**DOI:** 10.1038/s42003-021-01845-4

**Published:** 2021-03-17

**Authors:** Joseph M. Kelich, Harry Papaioannou, Emmanuel Skordalakes

**Affiliations:** The Wistar Institute, Philadelphia, PA USA

**Keywords:** Cancer, Senescence, Mechanisms of disease, Telomeres

## Abstract

The human CST complex composed of CTC1, STN1, and TEN1 is critically involved in telomere maintenance and homeostasis. Specifically, CST terminates telomere extension by inhibiting telomerase access to the telomeric overhang and facilitates lagging strand fill in by recruiting DNA Polymerase alpha primase (Pol α-primase) to the telomeric C-strand. Here we reveal that CST has a dynamic intracellular localization that is cell cycle dependent. We report an increase in nuclear CST several hours after the initiation of DNA replication, followed by exit from the nucleus prior to mitosis. We identify amino acids of CTC1 involved in Pol α-primase binding and nuclear localization. We conclude, the CST complex does not contain a nuclear localization signal (NLS) and suggest that its nuclear localization is reliant on Pol α-primase. Hypomorphic mutations affecting CST nuclear import are associated with telomere syndromes and cancer, emphasizing the important role of this process in health.

## Abstract (web summary)

Kelich, Papaioannou and Skordalakes investigate the intracellular localization dynamics of the mammalian CST, a protein complex critical for telomere maintenance and homeostasis. They find that the CST complex localizes to the nucleus in a cell cycle-dependent manner that requires binding by Pol α-primase and that mutations affecting its nuclear import are associated with human telomere syndromes and cancer.

## Introduction

Telomere length homeostasis is critical to cellular health^1^. Both abnormally short and long telomeres result in a predisposition to cancer and/or telomere syndromes^2,3^. Telomerase is responsible for adding TTAGGG repeats to chromosome ends to restore telomere length and protect the genome from gene erosion^4,5^. Aside from telomerase, two main protein complexes function in maintaining telomere homeostasis for mammalian cells: shelterin and CST^6^.

Shelterin contains six subunits, termed TRF1, TRF2, TIN2, RAP1, POT1 and TPP1. It serves to cap telomeres, preventing the single-stranded telomeric overhangs from being recognized as DNA damage^7^. In this way, shelterin prevents unwarranted DNA damage responses and deleterious chromosome fusions^8^. Apart from capping, shelterin is also a telomerase processivity factor^9^ as its subcomplex POT1–TPP1 recruits telomerase to telomeres for telomere replication^9,10^.

The CST complex is also critical in maintaining telomere homeostasis^11^. The human CST complex composed of CTC1, STN1, and TEN1^12^ has dual roles in mediating telomere homeostasis. CST terminates the telomerase extension reaction^13^ either by sequestering the telomeric overhang or via disruption of the TERT–TPP1 interaction^14^. CST also recruits DNA polymerase alpha primase (Pol α-primase) to the lagging telomeric strand to facilitate C-strand fill in, an essential process in finalizing double-stranded telomere replication^15^. Additionally, the CST complex removes G-quadruplex DNA structures that form within G-rich regions of the genome, especially at telomeres^16^. Still, new functions for the CST complex are being uncovered such as its role in replication stress^17^. Notably, numerous mutations within the CST complex have been found causative for telomere syndromes such as Coats plus and dyskeratosis congenita (DC) indicating the essential role of CST in telomere maintenance^18^.

While it is known that CST binds to single-stranded DNA and telomeric regions^19,20^ a gap in knowledge exists on where CST localizes within the living cell and whether it’s localization is a regulated process. It is worth noting that all currently understood functions of CST are based within the nucleus. CST function at telomeres and therefore nuclear localization is a critical aspect of CST biology. Proteins are made and are post-translationally modified in the cytoplasm. The shuttling of proteins between the cellular compartments is a highly regulated process that goes hand-in-hand with function. Thus, fully understanding the precise inner workings of CST cellular dynamics is critical to understanding the function of this complex. Mislocalization could lead to telomeric homeostasis defects or replication stresses and could be causative for disease.

Here we explore the in-depth intracellular dynamics of CST using live cell microscopy, cell-cycle synchronization, and biochemical techniques combined with CRISPR Cas9 knock-in labeling^21^. Our studies reveal that CST migrates between the cytoplasm and nucleus in a cell cycle-dependent manner. CST accumulates into the nucleus most readily during late S-phase, where after there is a departure from the nucleus prior to mitosis. We report that CST nuclear import is mediated through an importin alpha (importin α)-dependent mechanism even though there are no functional nuclear localization signals (NLS) found within the structures of CTC1, STN1, or TEN1. Instead, based upon mutational studies and shRNA knockdown, we propose that CST nuclear localization relies on Pol α-primase. Finally, our data reveals insights into how mutations within CST or PolA1 may result in a loss of CST function for certain telomeropathies and cancers.

## Results

### mCherry-CTC1 knock-in reveals a dynamic localization of CTC1 in live cells

**Fig. 1:**
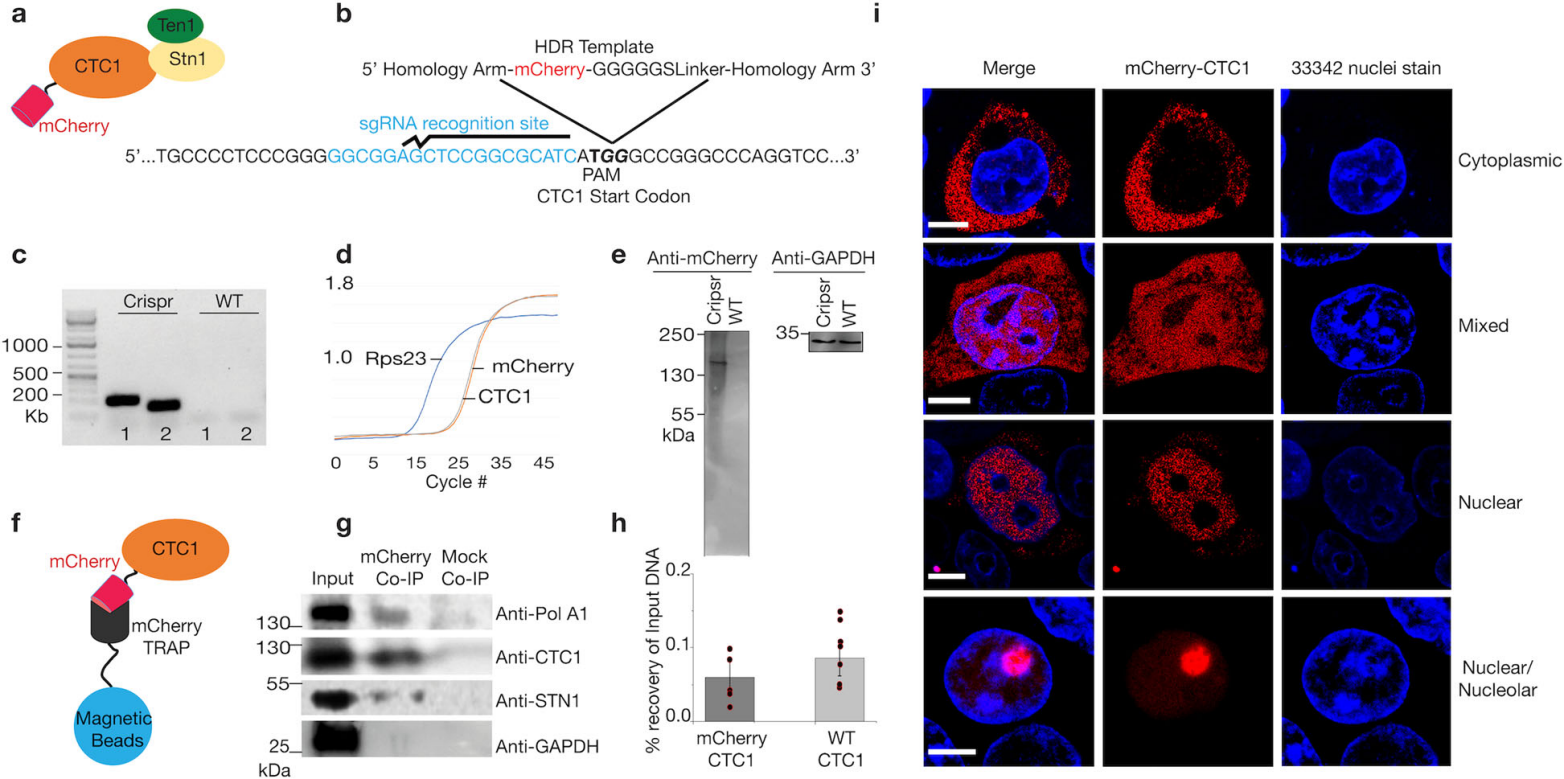
CRISPR Cas9 endogenous tagging of CTC1 reveals a dynamic intracellular localization. **a** An mCherry tag was genetically added to the N-terminal of CTC1. **b** The CRISPR Cas9 editing strategy is shown. A homology-directed repair template (HDR) was used in which the mCherry coding region was flanked by 1000 nucleotide homology arms coding for the CTC1 gene. The guide RNA utilized situates beside the ATG start codon to ensure N-terminal in-frame tagging of mCherry. **c** Genomic PCR of CRISPR edited and control cells was conducted using two sets of primers flanking the site of insertion of mCherry to confirm proper integration. **d** qPCR of reverse transcribed RNA obtained from the mCherry-CTC1 edited cell line quantifying mCherry and CTC1 signal. GAPDH is utilized as an endogenous control. **e** Western blot probing wild type HEK293T cells and CRISPR edited cells for mCherry reveals a single band of expected size. GAPDH was also probed for as a control. **f** Cartoon showing mCherry-Trap method to pull out CTC1 and associated proteins from cells. **g** Co-IP demonstrating mCherry-CTC1’s ability to associate with both PolA1 and STN1 but not GAPDH**. h** ChIP DNA recovery is shown for mCherry-CTC1 and wild type CTC1. **i** Confocal images of CRISPR-tagged mCherry-CTC1 cells. Within the same samples originating from single clones, mCherry CTC1 is seen as either predominantly nuclear, predominantly cytoplasmic, nucleolar, or a mixture of these conditions. 33342 was utilized to visualize chromatin.

In order to determine the localization of CTC1 in live cells, we utilized an endogenous fluorescent tagging strategy using CRISPR Cas9 on HEK293T cells. Guide RNAs were designed to induce a double strand break adjacent to the CTC1 start codon (Fig. 1a, b). In addition to this guide sequence, a homology-directed repair template (HDR) was designed with an mCherry sequence flanked by 1000 nucleotides of the CTC1 gene on both sides (Fig. 1b, Supplementary Fig. 1). By co-transfecting in the CAS9 enzyme with the guide RNA along with the HDR template, the endogenous homology-directed repair mechanism of the cell could recognize the template as homologous and repair the genome with the mCherry sequence added^21^. In order to enhance the efficiency of HDR, cells were treated with nocodazole 18 h prior to transfection^22^. This treatment increases the percentage of cells with 2N DNA thus increasing the chance of HDR to occur^23^. 72 h after transfection, mCherry expressing cells were sorted by flow cytometry and grown from single cells. Finally, cells were selected and confirmed for the mCherry knock-in at the N-terminal of CTC1 via western blotting, PCR, and sequencing (Fig. 1c–e, Supplementary Fig. 2). The ability of mCherry-CTC1 to form a complex with STN1 and to recruit Pol α-primase was then tested via mCherry trapping, using magnetic beads conjugated to mCherry-specific antibodies, and subsequent co-immunoprecipitations (Co-IPs) probing for endogenous STN1 and PolA1 (Fig. 1f–g)^24^. We found that mCherry-CTC1 associates with both PolA1 and STN1 but not GAPDH from cell lysates indicating that the fluorescent tag does not radically affect complex assembly or Pol α-primase recruitment and that the pull down is specific to CTC1 interacting proteins. Similarly, chromatin immunoprecipitation (ChIP) using a CTC1-directed antibody resulted in comparable recovery of genomic DNA between mCherry-CTC1 and WT cells (*p*-value > 0.1, one-tailed Student’s *t*-test) (Fig. 1h and Supplementary Data 1). Preliminary confocal microscopy experiments revealed that cells originating from singular parental clones demonstrate drastically different CTC1 localization from each other. Specifically, mCherry-CTC1 was found either in the cytoplasm, the nucleus, or as a mixture among different cells (Fig. 1i). Within cells containing CTC1 primarily in the nucleus, two subsets were found in which CTC1 was either sequestered within nucleoli or avoided the nucleoli nearly entirely (Fig. 1i). It should be noted that the majority of cells localized CTC1 within the nucleus. Preliminary widefield fluorescence imaging from a second knock-in clone produced indistinguishable results (Supplementary Fig. 3).

### CST complex localization is cell-cycle regulated

Telomere replication is cell cycle regulated with telomerase-dependent telomere extension occurring in early S-phase and lagging strand fill in late S-phase^25,26^. This knowledge led us to reason that CTC1’s intracellular locale might be controlled in a cell cycle-dependent manner. To test this hypothesis, we trapped mCherry-CTC1 and unedited HEK293T cells in various cell cycle stages using nocodazole, and aphidicolin treatments at specific time intervals. Aphidicolin halts cells at the G1/S border prior to DNA replication while nocodazole stops cell cycle progression at the onset of mitosis^27^. We then confirmed the cell cycle stages for the treated cells at distinct time points using propidium iodide flow cytometry analysis (Supplementary Fig. 4)^28^.

**Fig. 2:**
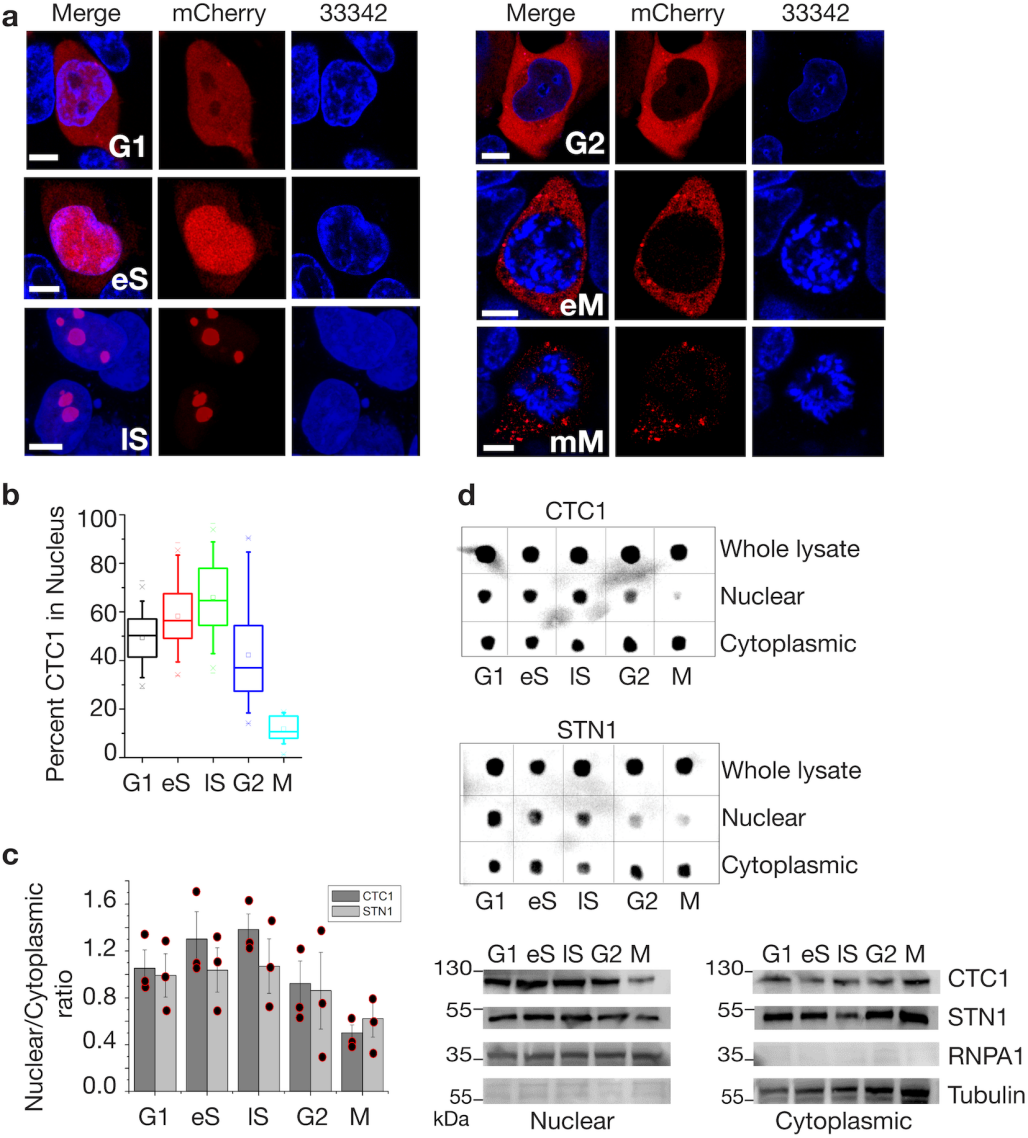
Localization of mCherry-CTC1 is cell cycle dependent. **a** Representative confocal images of mCherry-CTC1 cells for each cell cycle stage. eS early S, lS Late S, eM early mitosis (before nuclear envelope breakdown), mM mid mitosis. 33342 is Hoechst nuclear staining. **b** Quantification of nuclear percentage from microscopy images. Box plots are shown in standard format. Mean is an open dot, whiskers show the full range of data, the mean is represented by a horizontal line. *N* = 70, 52, 69, 39, and 13 for G1, eS, lS, G2, and M phases, respectively. Students *T*-test results are as follows. lS and G1, *t*-value = 8.24872, *p*-value is < 0.00001. lS and eS, *t*-value = 2.88675, *p*-value is 0.004624. lS and G2, *t*-value = 6.75264, *p*-value is < 0.00001. lS and M, *t*-value = 12.33461, *p*-value is < 0.00001. Scale bars are ~5 μm. **c** Quantification of the nuclear vs. cytoplasmic protein levels. **d** Dot blots are shown for nuclear and cytoplasmic fractions probing for CTC1 and STN1. Below, westerns are also shown to confirm efficient separation of cytoplasmic and nuclear fractions. Tubulin is a cytoplasmic marker while RNPA1 is a nuclear marker.

We employed two independent techniques to assess the localization of CTC1. We used confocal fluorescent microscopy as well as cell lysate fractionation analysis (of WT HEK293T cells without mCherry) to track the localization of CST throughout the cell cycle. As shown in Fig. 2a, b and Supplementary Data 1 there is an increase in nuclear CTC1 during S-phase peaking several hours after the initiation of DNA replication. However, we witnessed an en masse exit of CST from the nucleus prior to mitosis. For late S-phase we report a mean of 66% of intracellular CTC1 to be found within the nucleus. The percentage of nuclear CTC1 drops to ~10% by the start of mitosis. Nucleolar CTC1 was found most frequently during S-phase. It is worth noting that distinct localizations were not mutually exclusive for any cell cycle phase, potentially because chemical synchronization is not 100% efficient. Quantification examples for nuclear/cytoplasmic percentages for the confocal experiments can be seen in Supplementary Fig. 5. Cell cycle localization analysis of STN1 revealed increased nuclear import during S-phase and a dramatic decrease prior to mitosis as was seen for CTC1 (Fig. 2c, d).

### CST nuclear import is dependent on importin α-based mechanisms

**Fig. 3:**
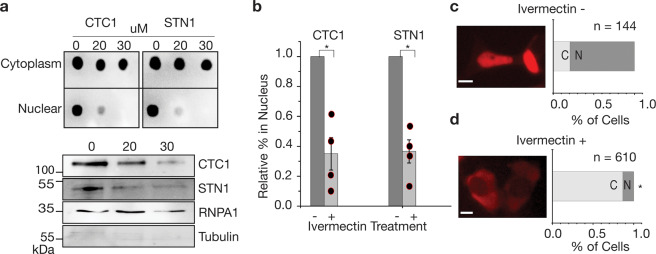
Nuclear import of CTC1/STN1 is reduced by Ivermectin treatment. **a** Cell lysate fractionation results for CTC1, and STN1 after being treated with Ivermectin at varying concentrations (μM). Dot blots as well as western blotting is shown to confirm the nuclear fractions. **b** Quantification of cell fractionation studies for ivermectin treatment. *p-value < 0.05. Student’s *t*-test results comparing control and ivermectin-treated cells are as follows. CTC1, *p*-value = 0.021392. STN1, *p*-value = 0.019171. **c**, **d** Epifluorescence microscopy analysis of mCherry-CTC1 or control cells after undergoing 20 μM ivermectin. Scale bar is ~5 μm.

We showed that CST migrates from the cytoplasm, where the protein is produced, to the nucleus where it is known to complete its functions. We also determined that CST mostly departs the nucleus and return to the cytoplasm prior to mitosis. Since CST shuttles between the cytoplasm and nucleus in a regulated manner, we set out to determine how this complex enters the nucleus. The human CST complex is ~188.5 kDa with CTC1, STN1, and TEN1 being 134.6, 42.1, and 13.8 kDa, respectively. Cargos destined for nuclear import >40 kDa in size cannot pass through nuclear pore complexes (NPCs) without the assistance of nuclear transport receptors (karyopherins)^29,30^. Importin α is one such transport receptor that in coordination with Importin beta (importin β) allows even large cargos to pass through NPCs to import into the nucleus^29,30^. We hypothesized that CST may be imported through an importin α-based mechanism and used drug treatments to inhibit importin α/β-dependent nuclear import. This importin mechanism is highly conserved and is responsible for the majority of nuclear import into the cell^31^. After treating the cells with varying concentrations of ivermectin (an inhibitor of importin α-mediated nuclear transport^32^), we found that there is a marked decrease in nuclear CST (Fig. 3 and Supplementary Data 1) from two independent techniques: epifluorescene microscopy of mCherry-CTC1 cells and cell lysate fractionation of WT HEK293T cells. This is consistent with our hypothesis that CTC1 is imported via an importin α/β-dependent mechanism. Nuclear import was drastically reduced for STN1 as well under all measured concentrations of ivermectin (Fig. 3a, b and Supplementary Data 1). At concentrations above 30 μM, cell viability was significantly inhibited with 40 μM causing 100% cell death within 24 h.

### CST mutants inhibit nuclear accumulation and diminish PolA1 binding

**Fig. 4:**
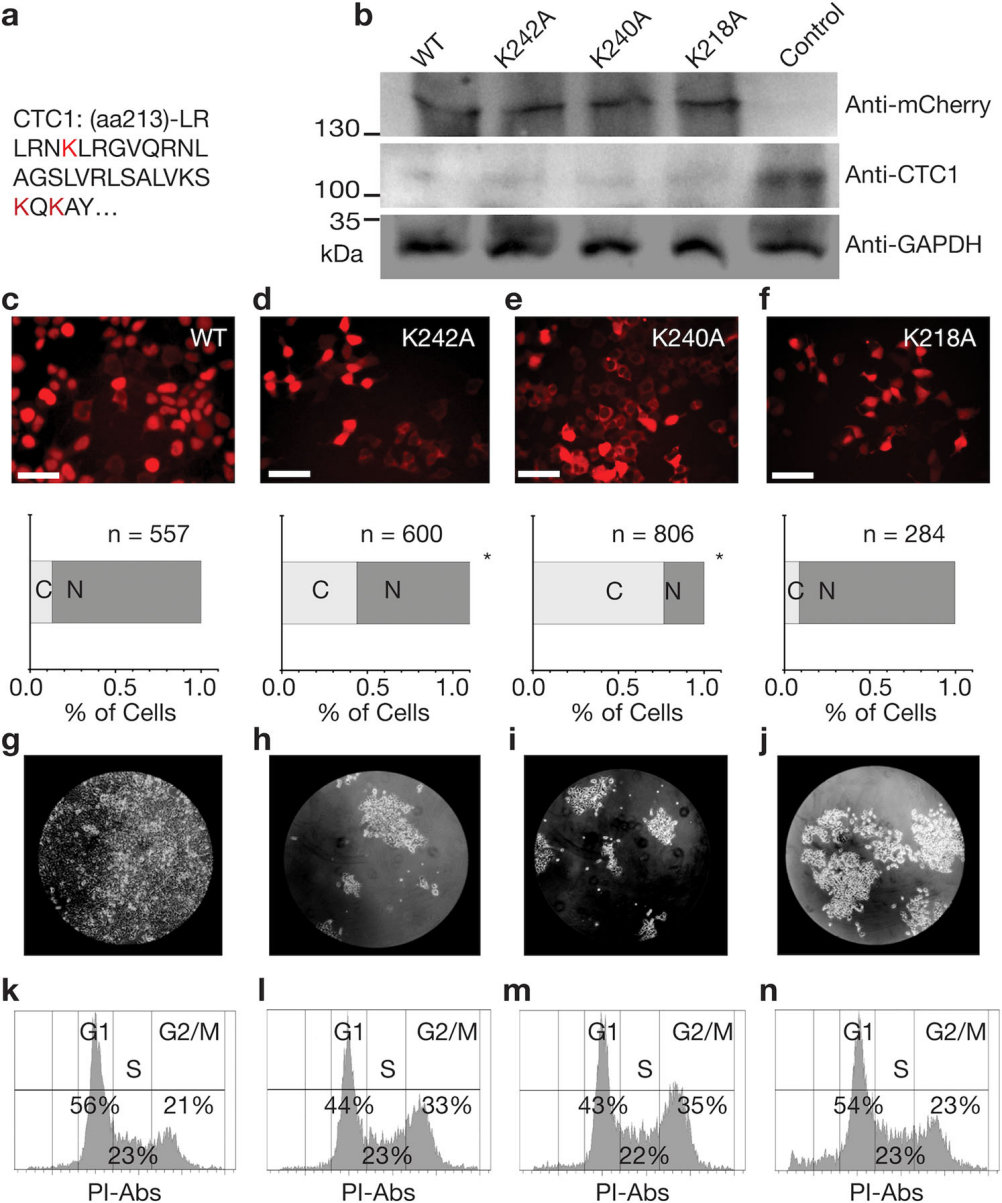
Mutant CTC1 constructs result in inhibited nuclear import and cell cycle defects. **a** The amino acids mutated are highlighted in red within the CTC1 amino acid sequence. **b** Western blotting results showing correct molecular weight and expression level of the mCherry-CTC1 construct after transfection into CTC1 knockdown cells. GAPDH was utilized as a loading control. **c**–**f** Epifluorescence microscopy analysis of mCherry-CTC1 mutants followed by quantification of the percentage of cells with predominately nuclear localization. *p-value < 0.001. When comparing K242A, K240A, and K218A to WT, using the binomial *Z*-test for proportions, the results are as follows. K242A, *z* = 11.1739 and *p* is <0.00001. K240A, *z* = 22.8747, and *p* is <0.00001. K218A, *z* = −2.087 and *p* is 0.03662. Scale bar is ~50 μm. **g**–**j** Cell growth results from one week follow up of 50,000 seeded cells showing growth defects for CTC1 mutants. All constructs were equally treated with CTC1 shRNAs and plasmid containing the wild type or mutant mCherry **k**–**n**. Propidium Iodide cell cycle analysis for the various mCherry-CTC1 cell lines.

To facilitate nuclear import, importin α, or one of its several isoforms, recognizes a NLS^33^. A classic NLS signal consists of a region rich in lysine/arginine, often located on a disordered portion of either the N or C-terminus of the cargo protein^31,33^. Classic examples are PKKKRKV located on the SV40 Large T-antigen and PAAKRVKLD found on c-Myc^34^. Importin α binds to NLSs, and then recruits importin β to the complex, which allows passage of the cargo through the nuclear pore complexes^35^. After screening the CST complex (CTC1, STN1, TEN1) genes manually and with several NLS predictors, including NLSdb^36^, SEQNLS^37^, NLStradamus^38^, and NLSmapper^39^, no classical NLS’s were identified. We selected a single NLS candidate of residues RLSALVKSKQK occurring within CTC1, based off homology to known NLS’s and proximity to disease mutations affecting nuclear import and PolA1 recruitment^14^ (Fig. 4a).

To determine if the predicted CTC1 NLS is essential for CST nuclear transport we did the following: We introduced each of the following single mutations K242A, K240A, and nearby K218A to the plasmid encoding mCherry-CTC1 and transfected them into HEK293T cells knocked down for endogenous CTC1 with shRNAs, as in our previous study^40^ (Fig. 4b). After generating stable cell lines through puromycin selection and flow cytometry, we analyzed these lines through epifluorescence microscopy (Fig. 4c–f and Supplementary data 1). We found that two mutants K242A and K240A significantly affect the concentration of nuclear CTC1 while the K218A mutation did not affect localization significantly. Dot blot cell fractionation experiment analysis also revealed that K242A and K240A result in less nuclear CST-based off probing for CTC1 and STN1 (Supplementary Fig. 6). Additionally, we selected another lysine residue located in an arginine/lysine cluster: RKPSK (residues 1162–1167) at the C-terminus to mutate because NLSs are usually found at the N or C-terminus and where the protein may be unstructured. This mutant was determined not to have any significant effect on nuclear localization (Supplementary Fig. 7). In addition to inhibiting nuclear import, mutations K242A and K240A also resulted in cell cycle progression defects. Cells carrying these mutations plated at equal seeding amounts were left to grow for one week. Figure 4g–j demonstrate the drastic growth inhibition for all mutants compared to WT mCherry-CTC1 cell lines. As seen in Fig. 4k–n, an increase in G2-arrested cells was found for cells containing mutations K242A and K240A. Percentage of G2 cells rises significantly from 21% for WT mCherry-CTC1 to 33% and 35%, respectively, for mutation K242A and K240A. This finding is consistent with previous studies showing G2 arrest occurring for CTC1 knockdown cell lines^41,42^.

**Fig. 5:**
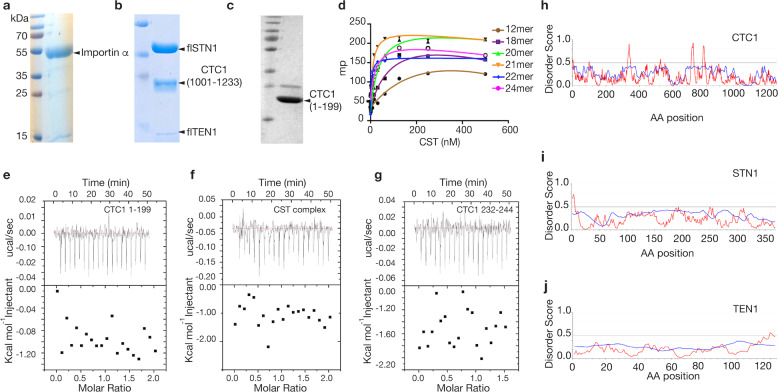
Importin α does not bind to CST. **a**–**c** Coomasie-stained protein SDS–PAGE gels are shown for **a** Importin α, **b** the functional CST complex, and **c** CTC1 1–199. **d** Fluorescence polarization data for CST binding to various size single-stranded telomeric repeats. **e**–**g** ITC data is shown between importin α and various CST constructs. **h**–**j** Disorder plots showing no significant disorder within CST. Values above 0.5 are deemed likely to be disordered. Iupred was utilized to model propensity for disorder^71^. Blue represents data from the Anchor2 algorithm, and red represents Iupred2 algorithm results.

To further determine whether the CST complex contains a functional NLS, we measured the ability of the assembled and active CST complex to bind importin α. We show in Fig. 3 that nuclear import of CST is importin α dependent, thus any functional NLS present within CST should bind importin α in vitro. Constructs we tested include the functional complex of full length STN1 (flSTN1), full length TEN1 (flTEN1) and CTC1 residues1002-end, the N-terminal portion of CTC1 (residues 1–199), and a synthetic CTC1 peptide of our candidate NLS (residues 232–244) (Fig. 5b–g). All proteins used in these studies were purified to homogeneity with >95% purity. We first showed that our assembled CST complex is active by performing fluorescence polarization (FP) assays to measure single-stranded telomeric DNA-binding ability. Our FP studies screening single-stranded DNA 2–4 telomeric repeats indicate that CST binds the 21mer (TTAGGG)3TTA with the highest affinity (*K*_d_ = ~21 nM) (Fig. 5d). Next, we tested all CTC1/CST constructs for binding to importin α using isothermal titration calorimetry (ITC). We expressed and purified a functional 6xHis-tagged human importin α construct lacking its autoinhibitory domain (aa70-end) (Fig. 5a). All assessed CST and CTC1 constructs displayed no evidence of binding to importin α. With this data we argue that human STN1, TEN1, and the N-terminal and C-terminal regions of CTC1 do not contain functional NLS signals (Fig. 5e–h). This is further supported by the lack of disorder within these three proteins (Fig. 5h–j)^43,44^.

**Fig. 6:**
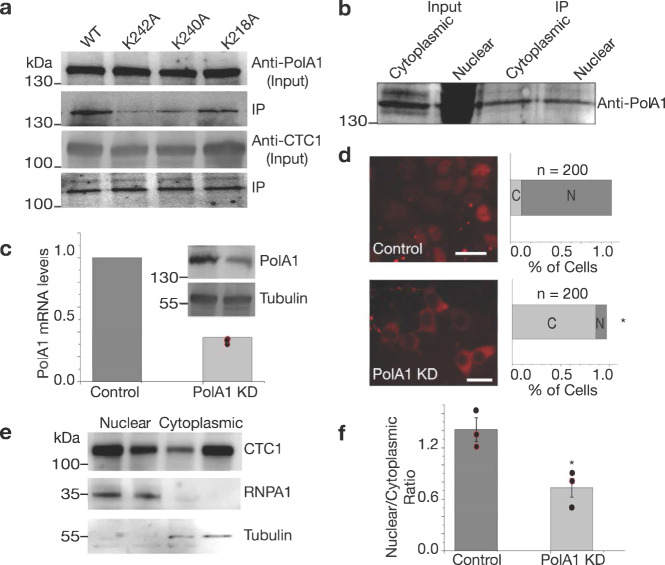
CTC1 mutants affect PolA1 binding and shRNA knockdown of PolA1 leads to less CTC1 nuclear accumulation. **a** Western blot data is shown for wild type and mutant CTC1 construct expressing cells after RFP-Trap was employed. IPs were then probed for PolA1, and CTC1. **b** Cell-fractionated Co-IP using RFP-beads incubated with nuclear and cytoplasmic extracts of WT HEK293T cells. **c** shRNA knockdown of PolA1 is quantified using reverse-transcribed DNA and qPCR as well as western blotting. Tubulin was utilized as loading controls. **d** Epifluorescence imaging of empty vector treated mCherry-CTC1 cells (control) and shRNAs targeting PolA1 (PolA1 KD). The value of *z* is −13.0997. The value of *p* is < 0.00001 (binomial *Z*-test for proportions). Scale bar is ~20 µm. **e** Cell lysate fractionation western blotting was performed on WT HEK293T cells treated with either empty vector or shRNAs targeting PolA1 demonstrating reduction of nuclear CTC1 in the presence of PolA1 knockdown. First lane of each fraction represents control cells, while the second lane of each fraction represents the knockdown cell lines. **f** Quantification of the results of three trials of the experiment shown in **e**. *p-value < 0.001. The obtained *p*-value comparing conditions is 0.0185 from Student’s *t*-test.

Despite not binding to importin α, the putative CTC1 NLS region still affected the nuclear localization of CTC1. As this region of CTC1 was previously shown to also affect PolA1 binding, we next tested whether these mutants can still bind PolA1 using mCherry bead-based Co-IPs probing for PolA1. As seen in Fig. 6a, K242A and K240A hinder PolA1 association with CTC1 in a manner similar to telomere syndrome-associated mutations A227V and V259M^14^. We additionally found that CTC1 is associated with PolA1 at similar amounts in both the cytoplasm and nucleus, despite PolA1 being significantly more abundant in the nucleus (Fig. 6b). All these findings led us to the hypothesis that PolA1–CST association is required for its nuclear localization. We next searched for a functional NLS on PolA1. By searching NLSdb^36^, we identified a putative NLS signal: KKSKKGR (residues 25–31) within the N-terminal domain of PolA1 (Supplementary Fig. 8c) that also meets the criteria of being located within a disordered region of the protein. To test functionality of this NLS, we expressed and purified the N-terminus of PolA1 1–338 (Supplementary Fig. 8a). We then tested this construct’s binding ability to importin α using ITC. We found that PolA1 1–338 binds importin α with a *K*_d_ of 14.2 ± 2.1 µM, which is comparable to what has been observed for NLS peptides binding to importin α such as that from SV40, pUL56, and transcription factor NIT-2^45–47^. This ITC data suggests that PolA1 contains a functional NLS at its N-terminus. To further validate whether the N-terminal putative NLS of PolA1 is required for nuclear import of PolA1 and CTC1, we performed mutagenesis replacing lysine and arginine residues of the putative NLS with alanine residues. Surprisingly, we found that none of the mutations significantly affected the ability of PolA1 to enter the nucleus (Supplementary Fig. 9).

### CST nuclear localization is dependent on POLA1

To further establish the importance of PolA1 on the nuclear localization of CST, we generated shRNA-mediated knockdown cell lines targeting PolA1. A total of seven shRNAs were tested in HEK293T cells of which two were found to efficiently knockdown PolA1 (Fig. 6c and Supplementary data 1). Stable cell lines were generated using puromycin selection using both WT HEK293T cells and mCherry-CTC1 cells. Remarkably, we show through both cell lysate fractionation of WT PolA1 knockdown cells and through epifluorescence microscopy of mCherry-CTC1 PolA1 knockdown cells that CTC1 nuclear localization is mostly lost (Fig. 6d–f and Supplementary data 1). To address the possibility that the loss in CTC1 localization may be caused from cell cycle defects caused from PolA1 knockdown we performed propidium iodide cell cycle analysis using flow cytometry for scramble shRNA control samples and shRNAs targeting PolA1. We found that HEK293T cells knocked down for PolA1 have increased G1 and S phase populations, while G2 populations decreased relative to control cells (Supplementary Fig. 10). These cell cycle defects however are most likely not the sole cause for the decrease in nuclear CTC1 as cytoplasmic CTC1 is primarily found during G2/Mitosis.

## Discussion

The human CST complex carries out several cellular functions critical to maintaining telomere length homeostasis^6,11^. Here we show that CST has a dynamic intracellular localization that is cell-cycle regulated. CST accumulates in the nucleus most significantly during S-phase, peaking at the end of DNA replication, before being pushed out of the nucleus en masse prior to mitosis. CST components, as all proteins, are generated in the cytoplasm but all known functions of CST occur within the nucleus. This emphasizes the importance of the nuclear import/export of CST and cell cycle regulation. Since prior to the mitotic phase CST is pushed out of the nucleus, the cytoplasm may function as a place to sequester CST when its canonical functions are no longer required. Other possibilities include CST having alternative functions in the cytoplasm. For example, p53 functions primarily in the nucleus in transcription regulation. However, this protein is exported to the cytoplasm in a MDM2-regulated manner to complete its moonlighting interactions with both microtubules and mitochondria^48^. Additionally, proteins are often exported at specific cell cycle time points to allow for degradation in the cytoplasm^49^. This is particularly important when the quantity of protein is tightly regulated or if high levels of protein turnover are required for cell health^50^.

We show that the nuclear import of CST is regulated by importin α. We maintain that this mechanism is likely indirect due to the absence of a functional NLS within CST. We did not identify an active NLS signal on CTC1, STN1, or TEN1 through bioinformatic or biochemical approaches. Interestingly, our CTC1 mutagenesis studies that inhibit CST nuclear localization also affected PolA1 binding to CST. We further show that PolA1 and CTC1 associate with one another in both the cytoplasm and nucleus of the cell and that the presence of PolA1 is required for CTC1 nuclear localization. Furthermore, knockdown of PolA1 resulted in less nuclear CTC1, demonstrating the reliance of CTC1 nuclear localization on this protein.

Although our studies focus primarily on CTC1, our data suggest that CST shuttles between the cellular compartments as a complex. This is supported by the fact that STN1 displayed near identical localizations to CTC1 throughout the cell cycle (Fig. 2c, d and Supplementary Data 1). Additionally, the nuclear localization of STN1 was shown to also be affected by ivermectin treatment and CTC1 mutations K242A and K240A in a comparable manner (Fig. 3, Supplementary Fig. 6). We also show by ITC that STN1 and TEN1 do not have their own classical NLS signal as they do not bind importin α (Fig. 5).

Unlike the punctuate and consistent telomeric localization of most Shelterin components, CST was found to have a dynamic localization within the cell^51^. We did not observe fluorescence signal restricted to telomeric sites. CTC1 in an unexpected manner, especially during late S-phase, localizes to nucleoli, which agrees with fluorescence imaging obtained for Arabidopsis CTC1^52^. This may be indicative of evolutionary conservation in localization and function of CST between plants and mammals. A similar localization pattern was also obtained for human TERT (hTERT) proteins^53^. The exact function of nucleolar hTERT remains unknown although some data suggests telomerase assembly may occur in this cellular locale^54^. It is possible there could be interactions between telomerase and CST within the nucleolus but thus far no direct interactions between CST and telomerase are known. Another potential function of nucleolar CST could be a sequestration point prior to nuclear export but after its functions in halting telomerase and C-strand fill in. Alternatively, nucleoli may function as compartments tightly regulating the amount of free CST in the nucleoplasm. Presumably, the amount of free CST in the nucleoplasm will control the percentage loaded onto telomeric sites.

CST shuttles between the nucleus and cytoplasm in a cell-cycle-dependent manner, it is likely that post-translational modifications, such as ubiquitination or sumoylation mediates this process as has been reported for other proteins^55,56^. The drop of nuclear CST prior to mitosis and the loss of chromosomal association through mitosis (Fig. 2a) indicates that CST most likely does not function in capping condensed chromosomes.

We note that previous studies looking into CST localization have not described any cytoplasmic localization for wild type CTC1^18,57^. These studies have, to our knowledge, all relied on cell fixation and immunostaining, which require cell membrane permeabilization and antibody-based detection. In these type of experiments, cytoplasmic proteins may be reduced due to membrane permeability^58^. Cell type differences also cannot be ruled out. It is possible that cancer cells or cells of different tissue origin may have different localization for CST.

CTC1 and STN1 were initially identified as Pol α-primase accessory factors from pull down experiments^59^. STN1 has been shown to associate with PolA2^60^, the regulatory subunit of Pol α. Here we show that CTC1 binds either directly or indirectly to PolA1 in both the cytoplasm and nucleus. It has previously been shown that CST recruits Pol α-primase to telomeric DNA to facilitate lagging strand fill in^26^. Here we report that CST nuclear localization is dependent on the catalytic subunit PolA1. Two possibilities exist for how CST is dependent on PolA1 for its nuclear localization: (1) PolA1 functions as a linker between importin α and CST to support its nuclear import or (2) PolA1 contributes to CST nuclear retention instead of nuclear import. We argue that the first instance is more likely. If PolA1 was only responsible for nuclear retention of CTC1 we would expect to find a functional NLS within at least one of the CST genes. This is because we have shown that Ivermectin treatment, which directly affects importin α-based nuclear import and not other karyopherins such as transportin, results in decreased CST nuclear localization^61^. We have not been able to identify any classical NLS within CST or any region of CST that interacts with importin α. We have also performed additional experiments knocking down PolA1 and found that CTC1 localization drastically changes with CTC1 localizing almost exclusively to the cytoplasm. Although we believe PolA1 functions as a medium between CST and importin α, we cannot rule out the possibility that an unknown protein component functions as this linker and that PolA1 is essential only to keep CTC1 in the nucleus. We initially hypothesized that an N-terminal putative NLS found on PolA1 serves as a link between CST and importin α. However, mutations in the NLS region did not affect PolA1 nuclear localization. We subsequently came across several publications suggesting that the putative N-terminal NLS on PolA1 may be dispensable for nuclear import^62,63^. It is also possible that a C-terminal NLS on PolA1 or NLS’s found on other subunits of the Pol α-primase complex may compensate for the loss of the putative N-terminal NLS^62^. This protein is essential for complete DNA replication and this function occurs within the nucleus. It would not be surprising if this protein had several methods of connecting with importin α to reach the nucleus. Further studies will need to establish the requirements for Pol α-primase nuclear import and how this complex collaborates with CST to facilitate proper nuclear localization. In this study, we have generated two mutations CTC1 point mutations (K242A an K240A) that resulted in less accumulation of CTC1 in the nucleus and diminished PolA1 binding (as revealed through Co-IP experiments). Based on recent Cryo-EM structures^44^, the region of CTC1 containing these mutations is solvent accessible and potentially available for Pol α–primase interactions.

While we aimed to focus this paper more on the cell cycle regulation and nuclear accumulation process of CST, we conducted a set of preliminary experiments to explore the mechanism responsible for CST nuclear export. Since nuclear CST was mostly lost prior to the onset of mitosis, CST nuclear export is most likely a tightly regulated process. We report a slight increase in nuclear CST upon Leptomycin B treatment that increased upon rising concentration of Leptomycin B. Leptomycin B is a specific inhibitor of CRM1-mediated nuclear export^64^ (Supplementary Fig. 11a–c). We also located two putative nuclear export signals (NES) within CTC1 (Supplementary Fig. 11d)^65^. This indicates that the nuclear export receptor CRM1 may play a role in this process. Further studies determining whether CST has functional NES’s will be needed to elucidate this mechanism further.

As described above, nuclear localization of CST is critical to all its known functions including telomerase termination, C-strand fill in, and G quadruplex resolution^6,11,15^. Inhibition of CST nuclear localization is likely to have catastrophic effects on cell and organismal health. Two mutations associated with the telomere syndromes Coats Plus and DC, A227V and V259M, were previously shown to halt nuclear localization and diminish PolA1 and PolA2 association with CST^14^. Two amino acid residues identified in our study affecting both nuclear import and PolA1 association lie between these two previously tested mutations. This suggests that this region of CTC1 is critical to Pol α-primase–CST association. This region is therefore also essential to the nuclear import/nuclear accumulation mechanism^14,57^. We also found that these import defective mutants result in G2 arrest and growth inhibition, similar to CTC1 knockdown experiments from other labs^14,41,42^. This indicates a general loss of function of CST for these mutations. This is not surprising as all known functions of CST occur within the nucleus^6,15,16^. There are ~30 cancer-associated and 8 telomere syndrome-associated mutations reported to date located within the PolA1 binding/nuclear localization domain of CTC1 (Supplementary Tables 1 and 2)^66^. We predict that these mutations may disrupt CST–Pol α-primase association and potentially nuclear localization, leading to a general loss of function for CST. One specific mutation found to be associated with DC: K242L*41 will most likely lose Pol α association and nuclear localization capabilities^67^. This provides one possible mechanistic understanding to telomere syndromes caused by CST mutations. Since CTC1 loss results in unregulated telomere length, fragile telomeres, and increased genomic instability, the CST cancer-associated mutations in Supplementary Table 1 may contribute to oncogenesis^12,42^.

## Methods

### Cell culture and plasmid preparation

For all cell lines, DMEM (Cellgro) was utilized supplemented with 1× penn/strep (Sigma) and 10% heat-inactivated FBS (Biowest). Culture was conducted at 37 °C at 5% CO_2_. For expression of mCherry-CTC1 through plasmid delivery, WT and mutant CTC1 constructs were cloned into the pEGFP-C1 vector with an mCherry N-terminal tag. HEK293T cells that were knocked down for endogenous CTC1 expression as in our previous publication^40^ were then transfected using a Gene Pulser (Biorad) to electroporate 4 μg of DNA using an exponential wave function. Stable cell lines were then generated through mCherry signal sorted flow cytometry using a MoFlo AstriosEQ (Beckham Coulter) system and selected further with puromycin at 1 μg/ml. To generate an in-frame mCherry knock-in at the CTC1 N-terminus, CRISPR Cas9 was employed using homology-directed repair modeled after previous studies^21,22^. gRNA constructs listed in a below section were cloned into the PX459 Cas9 expression vector. We designed an HDR template using ~1000 nt homology arms of the CTC1 gene flanking the mCherry sequence as seen in Supplementary Fig. 1 that was then cloned into the pUC vector for mammalian expression. Prior to transfection, HEK293T cells were synchronized into the mitotic phase for 18 h using nocodazole. Cells were then co-transfected through electroporation with 4 μg of each of the PX459 and HDR plasmids. Four days following transfection and initial puromycin selection, mCherry-expressing cells were sorted through flow cytometry and grown up from single cells. Sequence validation was later performed on cellular progeny to identify correct clones. For transient GFP-based expressions, GFP-PolA1 was cloned into a PCDNA.3 vector with an N-terminal GFP sequence. Lipofectamine 2000 was utilized using standard protocols. For cell cycle synchronization cells were either serum starved for 48 h, treated with aphidicolin (2ug/ml), or nocodazole (100 ng/ml) (Supplementary Fig. 4). Cells were then monitored hourly using propidium Iodide staining and flow cytometry analysis on a LSRII-14 color instrument. A representation for gating can be seen in Supplementary Fig. 12. Analysis was conducted with FlowJo software. To further distinguish between G2 and M phase, DNA condensation was assessed with microscopy. Ivermectin treatment was conducted utilizing product # I8898 from Sigma. Cells were incubated at concentrations ranging from 10 to 40 μM in DMEM for 1.5 h prior to analysis. 40 μM and up was found to be lethal for HEK293T cells. For all shRNA knockdown experiments pLKO vectors were utilized with puromycin selection conducted at 1 μg/ml. A random scramble sequence was used for all control lines.

### Live cell imaging

Two microscopy systems were used for taking images. For cell cycle regulation experiments, we utilized live cell confocal microscopy. Live cell confocal images measuring mCherry and Hoechst 33342 staining were taken with a Leica TCS SP8 white light laser scanning confocal microscope using 1 μm Z slices and presented as maximum projection images using ImageJ software. Virtual gating was employed to avoid overlap between fluorescent signals of different wavelength. Nuclear/cytoplasmic ratios were calculated using the ImageJ plugin Intensity_Ratio_Nuclei_Cytoplasm.ijm as in previous publications^68,69^. Examples for this type of analysis can be seen in Supplementary Fig. 5. For binary analysis of nuclear or cytoplasmic staining, epifluorescence wide field imaging was performed on a Nikon TE2000 inverted setup.

### Western blotting and co-immunoprecipitations

For western blot analysis of endogenous CST constructs standard immunoblot protocols were employed with the following antibody dilutions: anti-CTC1 antibody (PA5-24695, ThermoFisher, 1:1000 dilution), and anti-human STN1 antibody (HPA037924, Sigma 1:1000 dilution). For the mCherry-CTC1 mutants and mCherry knock-in experiments anti-mCherry (43590S, Cell Signaling Technology, 1:1000) was used. Anti-PolA1 (24799S, Cell Signaling Technology, 1:1000) was used to probe for PolA1. For controls GAPDH (2118S, Cell Signaling technologies) was used at 1:1000 dilution. For cell fractionating confirmation anti-hnRNPA1 (8443S), and anti-Tubulin (2148S) both from Cell Signaling Technology were utilized at 1:2000 dilution. For all western experiments anti-rabbit HRP (7074S, Cell Signaling Technology) was utilized as a secondary antibody. Detection of antibody signal was conducted with chemiluminescence that was activated using Luminata Forte Western HRP Substrate (Millipore). The signal was detected and developed with either a LAS-3000 scanner (Fuji), or an RGB Typhoon system (Fig. 1g) (Amersham).

Nuclear/cytoplasmic fractionation was performed as follows. 1 × 10^6^ cells were trypsinized, spun down, washed twice with PBS and incubated for 30 min with end-over-end rotation at 4 °C in cytoplasmic lysis buffer containing 50 mM Tris, 10 mM NaCl, 0.5% TritonX100, and 1 mM EDTA. Lysates were then centrifuged for 15 min at 13,000 rpm. The supernatant was removed and saved as the cytoplasmic fraction. The cell pellet was then washed twice with cytoplasmic lysis buffer (a small amount was first removed and checked via microscopy to ensure nuclei remain intact) and resuspended in an equal amount of RIPA buffer as to what was used for the cytoplasmic lysis step. The lysate was then tumbled end-over-end once again at 4 °C for 30 min. The lysate was then spun down and the supernatant was recovered as the nuclear fraction. All buffers were supplemented with protease inhibitor cocktail (P8340 Sigma). Equivalent volumes of each nuclear and cytoplasmic fractions were subsequently utilized for dot blot or western analysis.

Co-IPs were conducted using magnetic RFP-Trap beads (Chromotek) combined with a Dynamag (ThermoFisher) following manufacturer protocols. 8 × 10^6^ cells were utilized for all experiments using RFP-Trap. Mock-Co-IPs were conducted using WT HEK293T cells without mCherry present as negative controls.

### Chromatin immunoprecipitation

8 million HEK293T cells expressing either mCherry-CTC1 or WT-CTC1 were collected and fixed using 1% methanol-free formaldehyde. Fixation was allowed to occur for 5 min with gentle rocking before the reaction was halted with 0.125 M glycine. Cells were then spun down at 1,300 RPM for 5 min. Liquids were aspirated and cell pellets were washed 2× with PBS. Cells were then resuspended in ChIP buffer (150 mM NaCl, 1% Triton X-100, 5 mM EDTA, 10 mM Tris, 0.5 mM DTT). Sonication was then performed at 10 °C using an ultrasonicator (Covaris). DNA shearing was optimized to generate fragments 200–700 bp in length. Sonicated chromatin was then cleared at 14,000 RPM for 10 min. 10% of each sample is stored as input at this stage. mCherry and WT samples were incubated with 2 μg either non-specific IgG isotype control antibodies (3900, Cell Signaling Technologies), or CTC1 antibody PA5-24695, Thermo) and Protein A Dynabeads (10001D Thermo). Rotation was then conducted at 4 °C overnight. The next morning samples were stringently washed 2× with each of the following buffers (150 mM NaCl, 20 mM Tris, 0.5 mM EDTA, 5% sucrose, 0.2% SDS), (0.5% deoxycholic acid, 500 mM NaCl, 50 mM Hepes, 0.5 mM EDTA, 1% Triton X-100), and (0.5% deoxycholic acid, 250 mM LiCl, 1 mM EDTA, 0.5% NP-40, 10 mM Tris). Samples were then eluted with 1% SDS TE buffer at 65 °C. Proteinase K was then added and allowed to digest the sample for 3 h at 50 °C. Samples were finally purified with chloroform extraction followed by ethanol precipitation. Total DNA recovered was quantified with spectrophotometry for Inputs, IgG control samples and CTC1 samples. Data was normalized to the IgG control samples for all analysis.

### Protein expression and purification

All constructs were expressed in *E.coli* ScarabXpress *T7 lac* competent cells (Scarab Genomics) and grown to optical density of 0.6 prior to 1 mM IPTG (isopropyl-β-d-thiogalactopyranoside, Gold Biotechnology) induction. For expression of CTC1 1-199, PolA1 1-334, and CTC1 1002-end a pMocr expression vector was utilized^70^. Importin α, and TEN1 were expressed from N-terminal hexahistidine vectors, while STN1 was expressed in an HMK vector. All constructs were lysed in a buffer containing 25 mM Tris, pH 7.5, 1.0 M KCl, 1.0 M urea, 5% glycerol, 1 mM phenylmethylsulfonyl fluoride (PMSF) and 1 mM benzamidine via sonication. Each lysate was then initially purified via nickel–nitrilotriacetic acid (Ni–NTA) columns and buffer exchanged with 25 mM Tris, pH 7.5, 0.2 M KCl and 5% glycerol. Each construct was then further purified via HS and HQ (Poros) affinity columns. STN1 and TEN1 were co-cracked via sonication and purified as a complex with an additionally amylose column step prior to HS-HQ loading (New England Biotech). The resultant STN1–TEN1 complex was then incubated with purified CTC1 (1002-end) at 4 °C to allow for final CST complex formation. Complex formation was confirmed via amylose column binding (CTC1 does not have affinity for the amylose resin) and SDS–PAGE analysis. All tags were cleaved with TEV protease were applicable prior to experiments. Finally, purified proteins were passed through a Superdex S200 (GE Healthcare) column to remove any aggregates.

### Fluorescence polarization

FP DNA-binding assays were performed with an Envision Xcite Multilabel Plate Reader (Perkin Elmer). Reactions were carried out in 20 mM Hepes, pH 7.5, 100 mM KCl, 2 mM MgCl_2_, 1 mM EDTA, 2 mM DTT, 1 mg/ml BSA, 5% v/v glycerol, and 75 nM polyT50 competitor. The 12-24mer DNA probes (TTAGGG) were purchased with a 5′ 6-FAM label from Integrated DNA Technologies (IDT). Probe concentrations of 2.5 nM were used for all experiments. Reactions were incubated at room temperature for 30 min and performed in triplicate in light-proof optiplates (PerkinElmer). Probes were excited with 480 nm light and emissions measured at 535 nm. Milipolarization (mP) values were calculated through Envision software (PerkinElmer). All curves were fit with a one-site binding, nonlinear regression models using PRISM 5.0 (GraphPad Software, San Diego, CA, USA, www.graphpad.com).

### Isothermal titration calorimetry

Prior to ITC experiments, all prepared proteins were extensively dialyzed into a buffer containing 25 mM Hepes, pH 7.5, 0.1 M KCl, 5% glycerol, and 1 mM TCEP. Experimentation was performed on a MicroCal iTC200 (Malvern) and data analysis was conducted with Origin7 software (OriginLab Corporation). 20 μM of importin α was loaded into the cell for all experiments shown and 200 μM of injectant was dispensed in 2 μl increments. All experiments were conducted at 25 °C with 300 rpm spinning and 150 s spacing between injections.

### gRNA primers and primers for knock-in confirmation

The following primer pairs were cloned into the PX459 Cas9 vector. 5′CACCGCTCCGGCGCATCATGGCGGC and 5′AAACGCCGCCATGATGCGCCGGAGC. 5′CACCGGAGCTCCGGCGCATCATGG and 5′AAACCCATGATGCGCCGGAGCTCC

Primer pair 1 for knock-in confirmation:

(5′CACCATCGTGGAACAGTACG + 5′CCAGAGAGACAAGGCAGAGG)

Primer pair 2 for knock-in confirmation:

(5′CACCATCGTGGAACAGTACG + 5′CCCAGAGAGACAAGGCAGAG)

### shRNA and qPCR primers utilized

shRNAs: For CTC1 TRCN0000330094 hCtc1 (shCtc1) was obtained from the Sigma Mission shRNA library and used in our previous publication^40^. For PolA1 the following sequences were utilized: CCAGCTTGTATCGTTGCAGTA and CGCAATAAAGACAAGAGGAAT in an iBLAST vector. The following primers were utilized for qPCR: GAPDH (5′ATGGAAATCCCATCACCATCTT and 5′CGCCCCACTTGATTTTGG)

CTC1 (5′GTGATTGAACCAAGGACTCCAGAT and 5′CAGGCTGGCACCAGAACAC);

mCherry (5′CCCCGTAATGCAGAAGAAGA AND 5′TCTTGGCCTTGTAGGTGGTC)

PolA1 (5′GCCAGCAGAGGAAGTGAAAC and 5′CCCTTTTACCAATGGGAGGT) + (5′GCCAGGATGATGACTGGATT and 5′CTTTCCAGCACAAGCAATGA)

### Statistics and reproducibility

Appropriate statistical tests including binomial *Z*-test for proportions and Student’s *t*-tests were routinely utilized as described in the figure legends. All instances where conducted two-sided. Sample sizes with each *N* representing unique samples are also included in each figure legend. Individual data points are plotted where applicable. All error bars shown are the standard error of the mean.

### Reporting summary

Further information on research design is available in the Nature Research Reporting Summary linked to this article.

## Supplementary information

Supplementary Information

Description of Additional Supplementary Files

Supplementary Data 1

Reporting Summary

## Data Availability

All data generated or analyzed during this study are included in this published article (and its supplementary information files). Unprocessed blots can be seen in Supplementary Fig. 13. Supplementary Data 1 contains data corresponding to the Figs. 1h, 2b, c, 3b–d, 4c–f, and 6c, f.
