# Supplementary material for: Pol α-primase dependent nuclear localization of the mammalian CST complex
Source: Commun Biol. 2021 Mar 17;4:349. doi: 10.1038/s42003-021-01845-4 (PMC7969954; doi:10.1038/s42003-021-01845-4)
Supplement: Supplementary file 1 — Supplementary Information [file 42003_2021_1845_MOESM1_ESM.pdf]

Pol α-primase Dependent Nuclear Localization of the Mammalian CST Complex

Joseph M Kelich, Harry Papaioannou, Emmanuel Skordalakes*
The Wistar Institute, 3601 Spruce St, Philadelphia, PA 19104, USA.

*Correspondence and requests for materials should be addressed to Emmanuel Skordalakes (skorda@wistar.org).

GGGGCTTTGGTGGCATTACGAGGAAGCCCTTCTCTGCTTGGCAAGATCAGGGAGACTGAAAGATGC
TTAGAAGTCATCCACCAGGCGGGCTGTGTTTGAGTTCCTGAGCTCTGCTACCCCATCCATCTCAAGG
CCTGCGGGATGCCGGACAGGATGTCACTTAGGAGGGAGACGGAGGCTGGGCGCGGTGGCTCCCG
CCTGTAATCCGAGCACTTTGGGAGCCCGAGGCGGGCAGATCGCTTGAGCCCAGGAGTTCGAGACC
AGTCTGGGCAACATGGCGAAACCATGTCGCTACAGAAAAATACAAAAATTAGCCGGGTGTGGCGGT
CCAAGCCTGCGATCCCAGCTACTTGGTGGGCTGAGGTGGGAGGATCGCTTGAGCCCGGGAGGTCG
AGGCTGCAGTGAACCGAGATGGCGCCGCTGCACTCCAGCCTGGGCGATAGAGCGAGACCTCGTCT
CAAAAAAATCAGAGAGGGCCAGGGGCTGTGTTTCACGCCTGTAATCCCAGCACTTTGGGAGGCTGA
GGCGGGTGGATCACCTGAGGTCAGGAGTTTGAGACCAGCCTGGCCACATGGTGAAACCCCGTCTCT
ACTAAAAATACAAAAAAAAAAAAAAAAAAAAAAAATTAGCCGGGCGTGGTGGTGGCAGGTGCCTGTAA
TCCTAGCTACTCAGGAGGCTGAGGCCGGAAAATCCCTTGAACCTGGGAGGCGGGGGTTGCAGTGA
GCCGAGATCGCGCCACTGCACTCCAGCCTGGGCGACAGAACCAGGCTTCGTCTTAAAAAAAAAAAA
ATCAGAGAAAAGAGACAGGGGTGTCGGGAGTTGGGATGGTGCACCGAGGCGCCTCGCGGAGTCTC
TAGGAAGCGAGGGGGTGAGCAGCGAGAAGGAGCTGGAGGTGGCGAGGAAACCCGCTCACCGGTG
ACGTCACCGAGCGCCCCCCCACCCCCATCCCCACCCCCAAGCGAGCACCTGCCCCTCCCGGGGGC
GGAGCTCCGGCGCATCATGgtgagcaagggcgaggaggataacatggccatcatcaaggagttcatgcgcttcaaggtgcacatg
gagggctccgtgaacggccacgagttcgagatcgagggcgagggcgagggccgcccctacgagggcacccagaccgccaagctgaaggtg
accaagggtggccccctgcccttcgcctgggacatcctgtcccctcagttcatgtacggctccaaggcctacgtgaagcaccccgccgacatcccc
gactacttgaagctgtccttccccgagggcttcaagtgggagcgcgtgatgaacttcgaggacggcggcgtggtgaccgtgacccaggactcctcc
ctgcaggacggcgagttcatctacaaggtgaagctgcgcggcaccaacttcccctccgacggccccgtaatgcagaagaagaccatgggctggg
aggcctcctccgagcggatgtaccccgaggacggcgccctgaagggcgagatcaagcagaggctgaagctgaaggacggcggccactacga
cgctgaggtcaagaccacctacaaggccaagaagcccgtgcagctgcccggcgcctacaacgtcaacatcaagttggacatcacctcccacaa
cgaggactacaccatcgtggaacagtacgaacgcgccgagggccgccactccaccggcggcatggacgagctgtacaagGGtGGtGGtG
GtAGTGCGGCTGGCCGGGCCCAGGTCCCTTCCTCCGTGAGTGCTGCTATTACGTCTCTTGTTCCTTT
TTTCTTGTTTGTCACAGATACTCTTTCTCTTCCTATTTCCTCTGCCTTGTCTCTCTGGGCTCGCTGGCC
GCGTCCGGCCCATGGGTGAGGGGGTTGTGAGTCGGTCCGCTCCTACCTCTTACTGGTTCTGCGAAT
GTTTATTCCCTCATTCACGCGGCGCCCGCTGTGAGCCTTAAACTGGTCTCCGTTCACTCTCTAGGGT
TTAATCCTGGTGGGAAACCAGGAGCAGGTTAATGGGGGAACTATGGGGCTGTGGGTTCCTGGAGAA
GACCCTTGACGGAGCTGTAGGGGAAAAGGGCAACCATAATAAGTAAAAGCGTCTGAGACCCGAAGT
GCGTGTAGAAGGCAAAAAGCGCCGAAAGGGGCTGGGTGTGGTGGCTCACGCTTGTAATCCAGCACT
GTGGGAGGCGGAGGCGGGCGGATTACTTGAGGTCAGGAGTTCGAGACCAGCCTGACCAACATGGT
GAAACCTCATCTCTACTAAAAACACAAAAATTAGCCGGGCGTGGTGGCGCGCACCTGTAATTCCAGC
TACTCAGAAGGTTGAGGCAGGAGAATCGCTTGAACCGGGTAGACGGAGGCTGCAGTGAGCTGAGAT
TGTGCCACAGCACTCCAGCCTGGGCAACAGAGCGAGACTCTGTCAAAAAAAAAAAAAAAAAAAAAAA
AGCGCCGGAAGGGGTTTCAGGACGGAAGCAAACACATATTCAAAGGCAGGAGGATAGAGCAGGTGT
CTGAAAAACTCAAAAGTTCAGTTGGGTGGTGGTGGAGGGGTCAGAGCTGACAGTTTTGTACCTCTTG
CTAAGCAGCGACTTTAACATGAGGGTAATTGGGTGTGGTGGCACACGCCGGTAATCCCAGCTGCTC
AGGAAGCTGAGCAGGGAACCACGGAAGTGGAGGTTGCAGTGAGCCGAGATCGCGCCACTGCACTC
TAGCCTGGACGACAGAGCCAGA

Red = mCherry

Yellow = Linker

Black = CTC1 gene

Supplementary Fig. 1. HDR Template sequence for CRISPR Cas9 mCherry knock-in at the CTC1 locus.

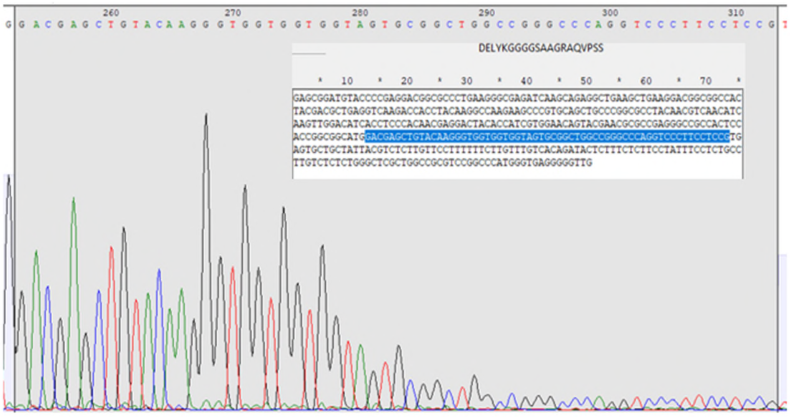

Supplementary Fig. 2. Sequencing data obtained from genomic amplification of mCherry-CTC1 CRISPR knock-in cells. Translation is highlighted to shows amino acids for the end of the mCherry followed by a Glycine-Serine linker and then the first exon of the CTC1 protein.

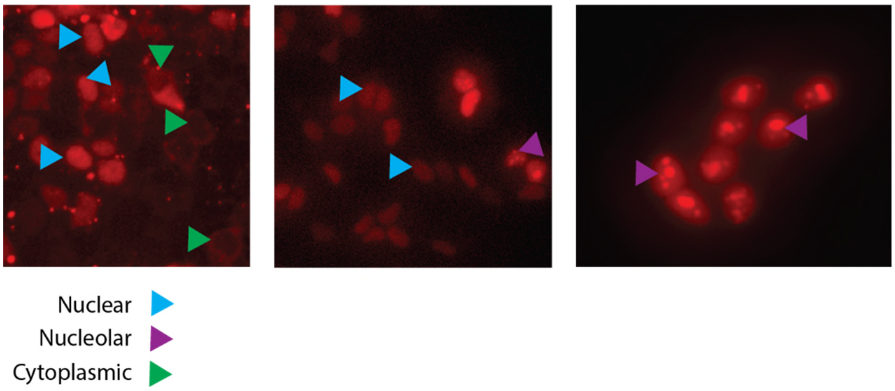

Supplementary Fig. 3. mCherry-CTC1 localization in a second mCherry-CTC1 knock-in clone. Widefield epifluorescence imaging of cells derived from a single parental clone show nuclear, cytoplasmic, and nucleolar localization for mCherry-CTC1.

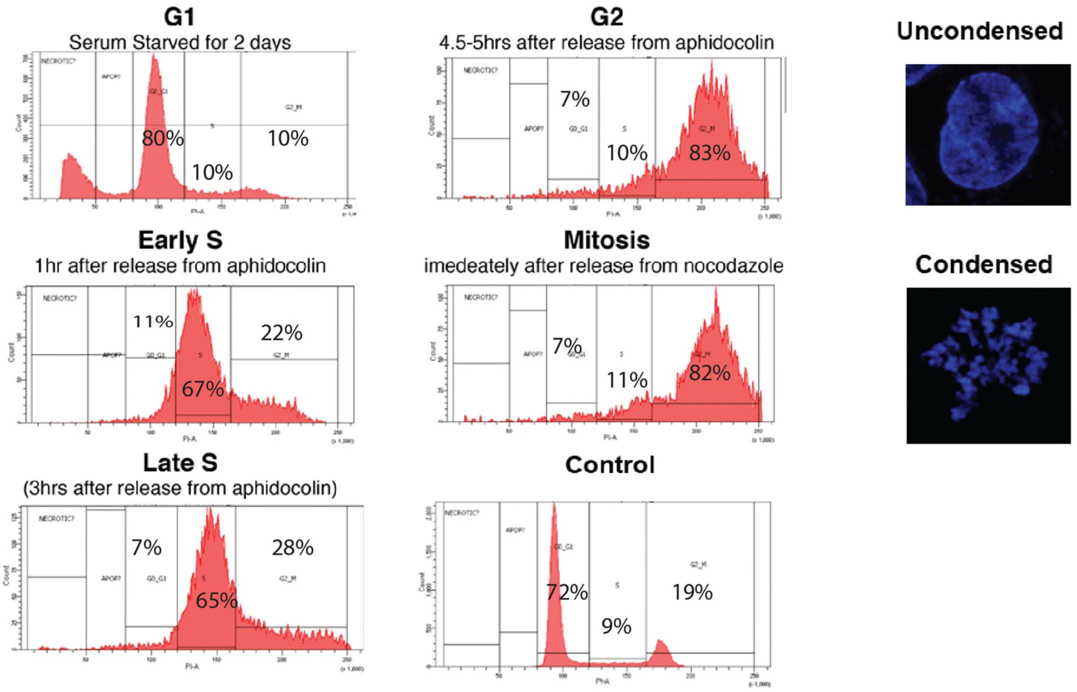

Supplementary Fig. 4. Cell cycle synchronization of mCherry-CTC1 HEK293T cells. Serum starvation, aphidicolin and nocodazole were employed to synchronize cell in the various cell cycle stages. Propidium Iodide flow cytometry was utilized to determine respective cell cycle staging. Condensation of chromatin as shown in blue was further utilized to distinguish G2 from Mitotic cells. No necrotic or dying cells were included in further analysis.

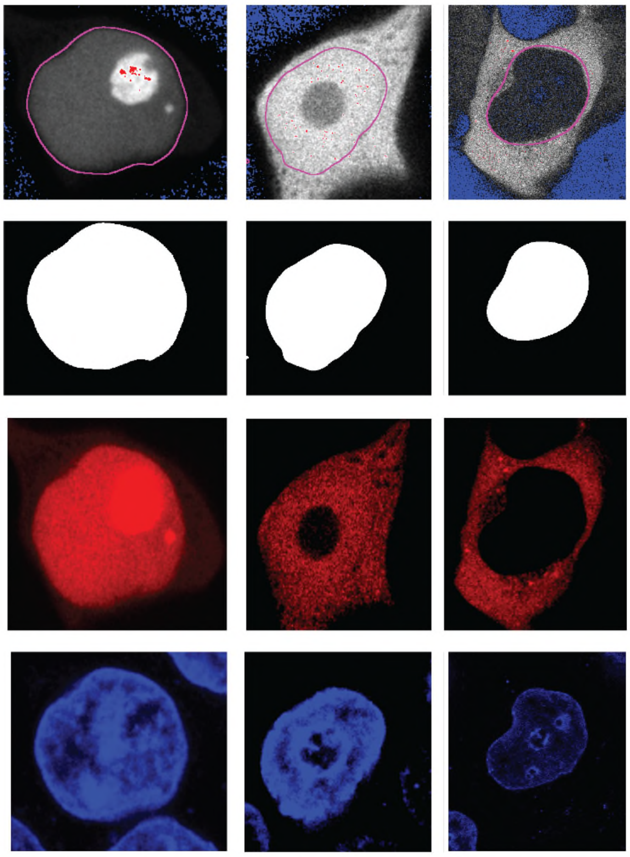

icn factor	% nuclei	% cytoplasm	av. nuclei intensity	av. cytoplasm intensity
7.2262	87.8438	12.1562	898.9886	87.3536
2.4105	70.6789	29.3211	101.8499	80.2793
0.2256	18.41	81.59	544.6233	1214.2913

Supplementary Fig. 5. Quantification method for Nuclear/Cytoplasmic ratio. Nuclear/cytoplasmic ratios were calculated using the ImageJ plugin Intensity_Ratio_Nuclei_Cytoplasm.ijm[1,2]. Three cells showing drastically different localizations for mCherry-CTC1 are shown with corresponding percentages in the nucleus and cytoplasm.

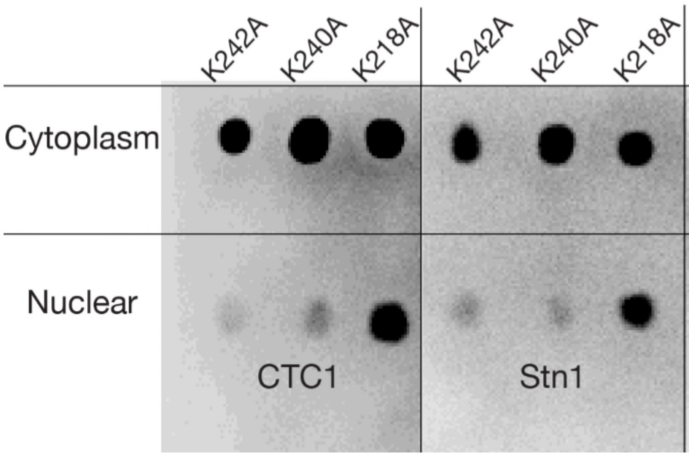

Supplementary Fig. 6. Mutant CTC1 constructs also inhibit nuclear import of STN1. Cell lysate fractionation was employed to split mCherry-CTC1 mutant HEK293T samples into cytoplasmic and nuclear fractions. K242A and K240A significantly diminish nuclear STN1 and CTC1 while K218A does not.

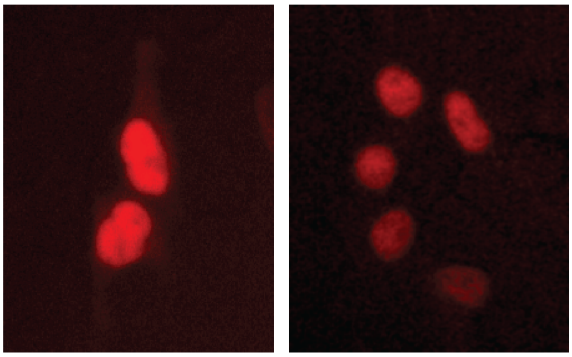

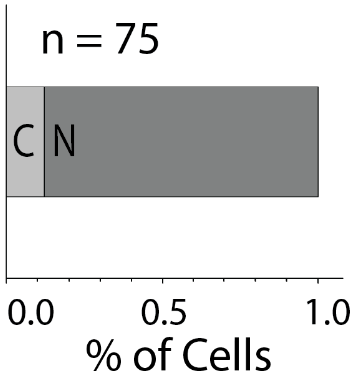

Supplementary Fig. 7. CTC1 Lysine 1164 is not critical to nuclear localization of CTC1. Lysine 1164 was mutated to an alanine on an mCherry-CTC1 expression plasmid and transfected into CTC1 knockdown cells. Fluorescence microscopy performed 48 hours later displays no inhibition of nuclear localization.

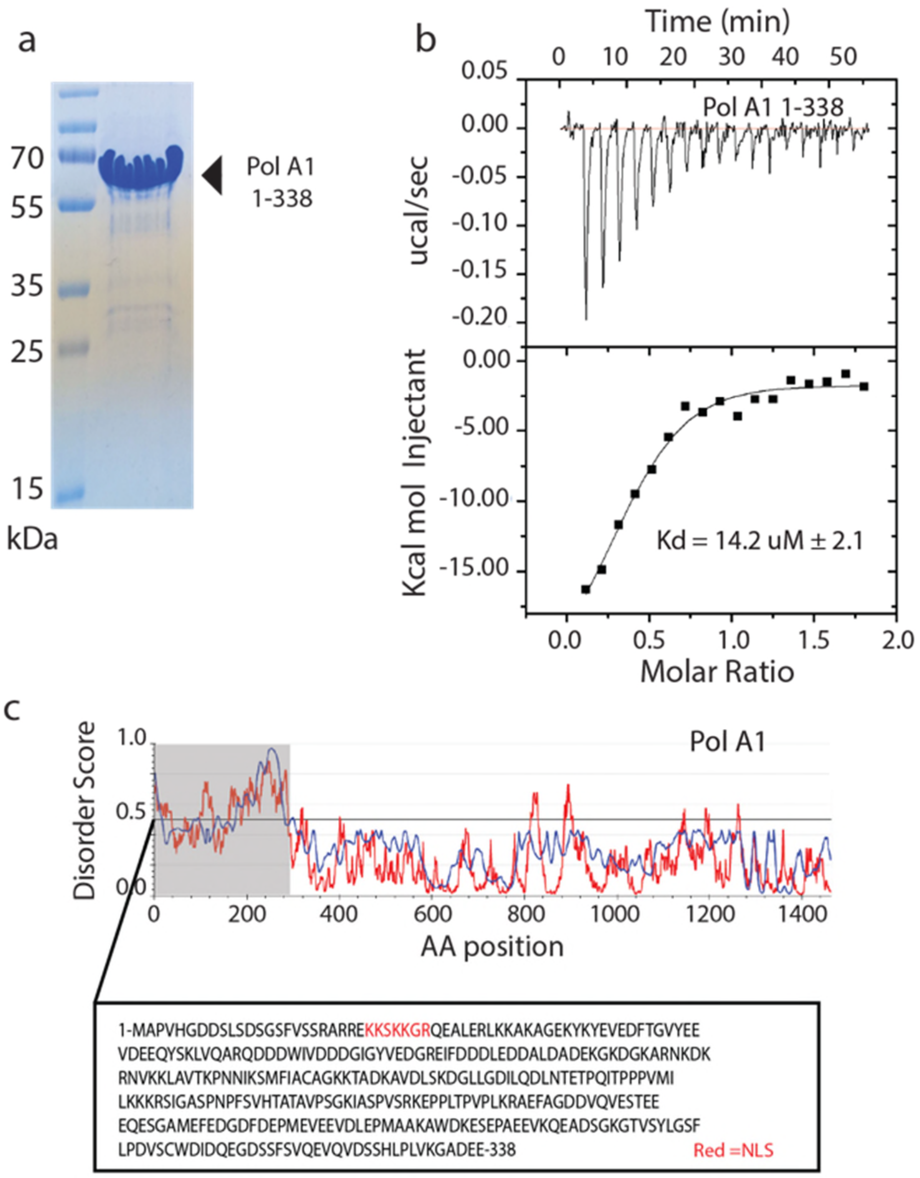

Supplementary Fig. 8. N-terminal tail of Pol A1 binds to importin α in vitro. **a.** SDS-PAGE analysis shows purified Pol A1 (1-338). **b.** ITC binding curve shows that importin α binds to Pol A1 with micromolar affinity. **c.** Disorder plot highlights how the N-terminus of Pol A1 is disordered. Values above 0.5 are deemed likely to be disordered.

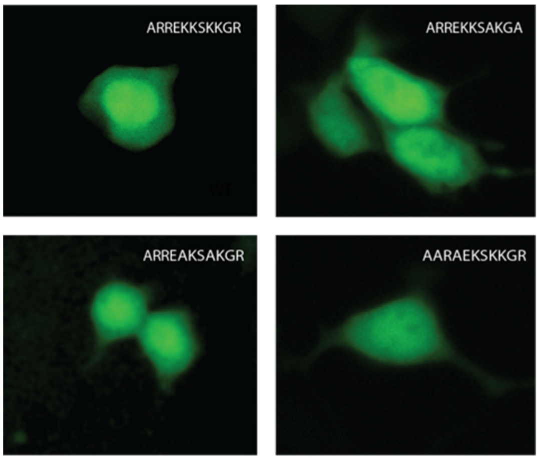

Supplementary Fig. 9. GFP-Pol A1 NLS mutants do not significantly affect Pol A1 nuclear localization. Widefield epifluorescence images were taken of GFP-Pol A1. Top left panel represents GFP-Pol A1 containing the wild type putative N-terminal NLS. Three other mutants are shown all with a localization within the nucleus.

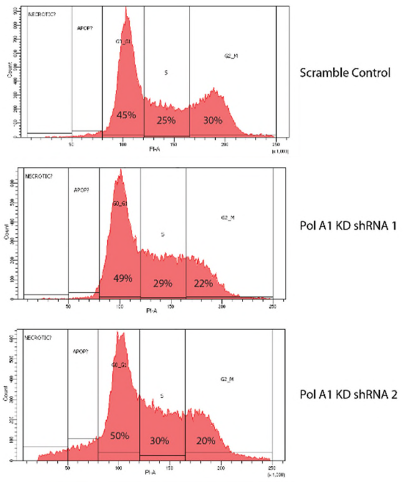

Supplementary Fig. 10. Pol A1 knockdown results in minor cell cycle defects in HEK293T cells. Propidium Iodide cell cycle analysis shows increase in G1 and S phases for Pol A1 knockdown cells compared to non-targeting control shRNAs.

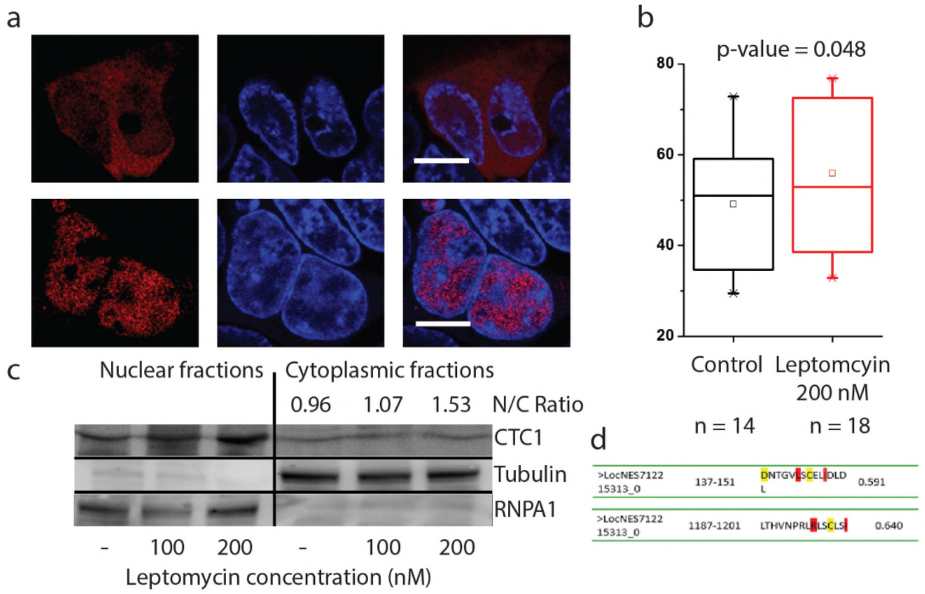

Supplementary Fig. 11. Leptomycin B treatment of mCherry-CTC1 cells. a. Confocal images showing daughter cells at the end of telophase for both control and leptomycin treated cells. Scale bar is ~5 um. More CTC1 is witnessed within the nucleus of the new daughter cells for leptomycin treated cells compared to control. b. Quantification of Nuclear/Cytoplasmic ratio of cells imaged through confocal microscopy shows a modest increase in nuclear CTC1. c. Nuclear/cytoplasmic fractionating western blots are shown for wild type HEK293T cells treated with leptomycin at varying concentrations. An increase in nuclear fraction of CTC1 is seen with increasing Leptomycin B concentration. d. Locnes was utilized to identify two potential nuclear export signals (NES) within CTC1[3]. Red indicates high conservation, yellow indicates minor conservation in amino acid reside composition between mammals.

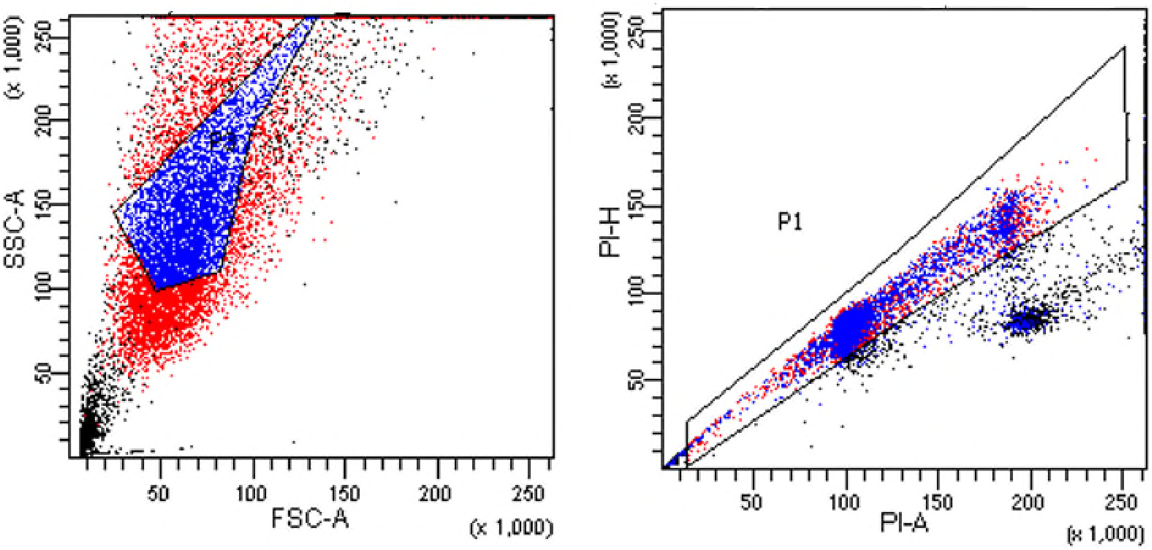

Supplementary Fig. 12. Flow cytometer gating example. A sample gating of cells is shown. All experiments used the same gating scheme. Cells deemed apoptotic/necrotic were excluded from analysis. mCherry expressing cells were deemed to be over 80% for mCherry based sorts and this number reaches 100% for propidium Iodide stained cells. >10,000 cells were used for all plots shown in the manuscript.

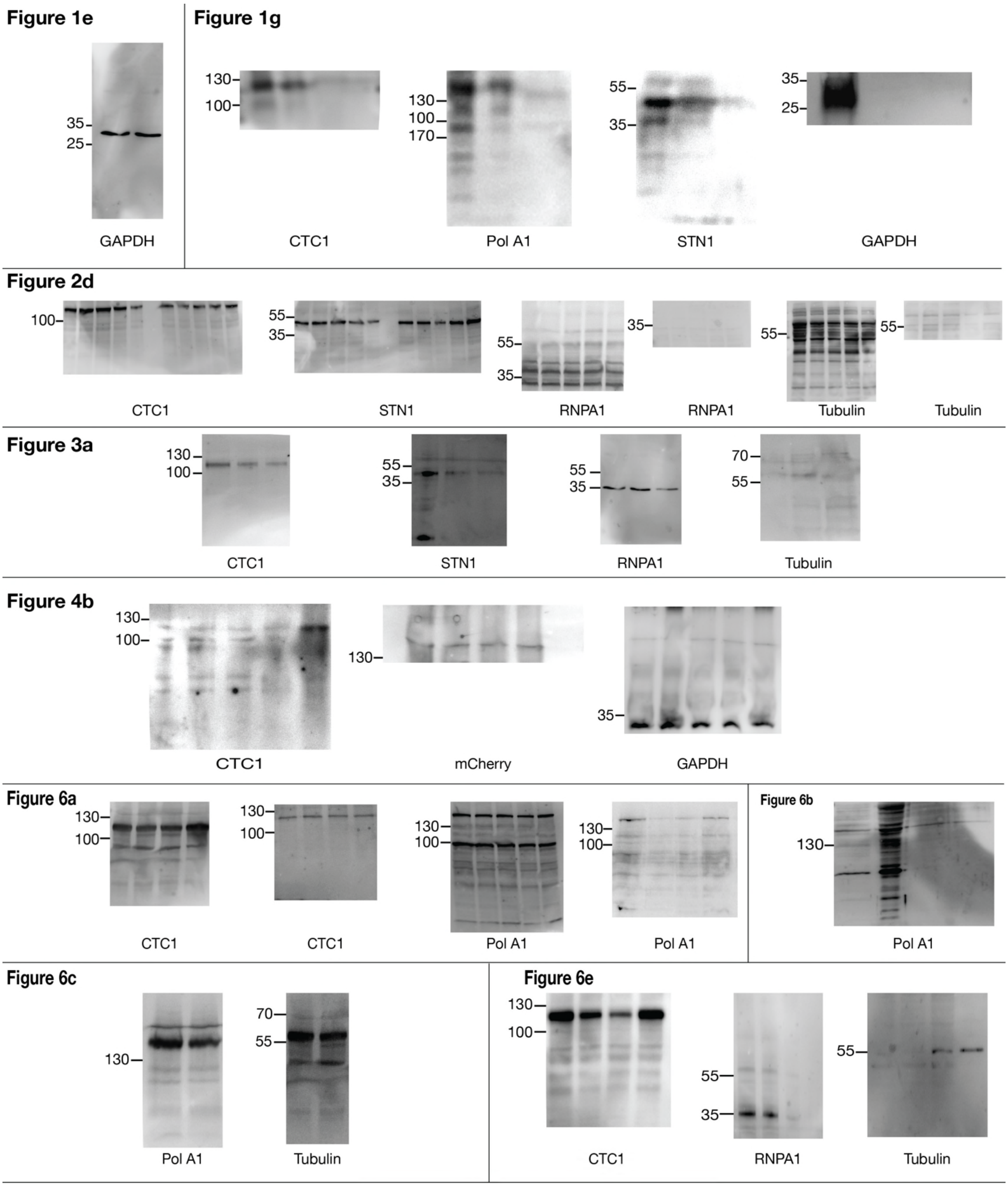

Supplementary Fig. 13. Unprocessed western blots. Blots included in the main text are shown as uncropped and unprocessed. All numbers shown represent kDa. Below each image is the target protein probed. Antibodies utilized are listed in the main text Methods section.

Amino Acid #	Reference AA	Replacement AA	Disease	Source
216	R	K	DOID:0070003 / blastoma	COSMIC
216	R	K	DOID:3571 / liver cancer	ICGC
223	Q	H	DOID:11054 / bladder cancer	TCGA
223	Q	L	DOID:1324 / lung cancer	COSMIC
225	N	K	DOID:3571 / liver cancer	ICGC
227	A	P	DOID:1324 / lung cancer	COSMIC
228	G	R	DOID:363 / uterine cancer	TCGA
233	L	F	DOID:1324 / lung cancer	COSMIC
241	Q	H	DOID:363 / uterine cancer	TCGA
245	F	L	DOID:3571 / liver cancer	ICGC
249	L	H	DOID:363 / uterine cancer	TCGA
253	H	Q	DOID:3571 / liver cancer	ICGC
253	H	Y	DOID:9256 / colorectal cancer	ICGC
254	P	Q	DOID:3571 / liver cancer	ICGC
259	V	G	DOID:1324 / lung cancer	COSMIC
263	V	M	DOID:10534 / stomach cancer	COSMIC
263	V	M	DOID:11934 / head and neck cancer	TCGA
264	Q	L	DOID:1324 / lung cancer	COSMIC
267	A	V	DOID:363 / uterine cancer	TCGA
270	V	A	DOID:363 / uterine cancer	TCGA
272	H	Q	DOID:3963 / thyroid carcinoma	COSMIC
276	R	W	DOID:1909 / melanoma	TCGA
285	E	K	DOID:0070003 / blastoma	COSMIC
285	E	K	DOID:3571 / liver cancer	ICGC
295	R	H	DOID:2994 / germ cell cancer	COSMIC
295	R	H	DOID:2394 / ovarian cancer	TCGA
316	Q	L	DOID:1324 / lung cancer	TCGA
317	E	K	DOID:5041 / esophageal cancer	ICGC
319	E	K	DOID:1909 / melanoma	TCGA
330	D	A	DOID:11054 / bladder cancer	ICGC
216	R	K	DOID:0070003 / blastoma	COSMIC
216	R	K	DOID:3571 / liver cancer	ICGC
223	Q	H	DOID:11054 / bladder cancer	TCGA
223	Q	L	DOID:1324 / lung cancer	COSMIC
225	N	K	DOID:3571 / liver cancer	ICGC
227	A	P	DOID:1324 / lung cancer	COSMIC
228	G	R	DOID:363 / uterine cancer	TCGA
233	L	F	DOID:1324 / lung cancer	COSMIC
241	Q	H	DOID:363 / uterine cancer	TCGA
245	F	L	DOID:3571 / liver cancer	ICGC
249	L	H	DOID:363 / uterine cancer	TCGA
253	H	Q	DOID:3571 / liver cancer	ICGC

Supplementary Table 1. Cancer-associated mutations within CTC1 and Pol A1

CTC1 Telomere Syndrome-Associated Mutations Within Pol A1/Nuclear Import Domain				
Amino Acid #	Reference AA	Replacement AA	Disease	Source
227	A	V	cerebroretinal microangiopathy	Polvi, et al, 2012
241	G	Stop	Coats plus	Anderson et al, 2012
241	G	Stop	dyskeratosis congenita	Walne et al, 2013
242	K	L Frame shift	Coats plus	Anderson et al, 2012
242	K	L Frame shift	cerebroretinal microangiopathy	Polvi et al, 2012
242	K	L Frame shift	Dyskeratosis Congenita	Keller et al, 2012
242	K	L Frame shift	dyskeratosis congenita	Walne et al, 2013
247	L	P	dyskeratosis congenita	Walne et al, 2013
259	V	M	Coats plus	Anderson et al, 2012
259	V	M	Norrie disease, cerebroretinal microangiopathy	Romaniello et al, 2012
278	G	V	dyskeratosis congenita	Walne et al, 2013
278	G	V	Coats plus	Bisserbe et al, 2015
281	Y	H	Coats plus	Bisserbe et al, 2015
287	R	Stop	Coats plus	Anderson et al,2012

Supplementary Table 2. Telomere Syndrome-Associated Mutations within CTC1

Supplementary References

1 Grbeša, I. *et al.* Mutations in S-adenosylhomocysteine hydrolase (AHCY) affect its nucleocytoplasmic distribution and capability to interact with S-adenosylhomocysteine hydrolase-like 1 protein. *European journal of cell biology* **96**, 579-590 (2017).

2 Schacke, M. *et al.* PARP-1/2 Inhibitor Olaparib Prevents or Partially Reverts EMT Induced by TGF-β in NMuMG Cells. *International journal of molecular sciences* **20**, 518 (2019).

3 Xu, D. *et al.* LocNES: a computational tool for locating classical NESs in CRM1 cargo proteins. *Bioinformatics* **31**, 1357-1365 (2015).
