# Supplementary material for: Pol α-primase dependent nuclear localization of the mammalian CST complex
Source: Commun Biol. 2021 Mar 17;4:349. doi: 10.1038/s42003-021-01845-4 (PMC7969954; doi:10.1038/s42003-021-01845-4)
Supplement: Supplementary file 2 — Description of Additional Supplementary Files [file 42003_2021_1845_MOESM2_ESM.pdf]

Description of Additional Supplementary Files

File Name: Supplementary Data 1

Description: Supplementary Data 1 contains data corresponding to the figures Fig. 1h, Fig. 2b, Fig. 2c, Fig. 3b-d, Fig. 4c-f.and Fig. 6c and f.
