# Supplementary material for: Pol α-primase dependent nuclear localization of the mammalian CST complex
Source: Commun Biol. 2021 Mar 17;4:349. doi: 10.1038/s42003-021-01845-4 (PMC7969954; doi:10.1038/s42003-021-01845-4)
Supplement: Supplementary file 4 — Reporting Summary [file 42003_2021_1845_MOESM4_ESM.pdf]

nature research

Corresponding author(s): Emmanuel Skordalakes

Last updated by author(s): 1/28/2021

Reporting Summary

Nature Research wishes to improve the reproducibility of the work that we publish. This form provides structure for consistency and transparency in reporting. For further information on Nature Research policies, see our Editorial Policies and the Editorial Policy Checklist.

Statistics

For all statistical analyses, confirm that the following items are present in the figure legend, table legend, main text, or Methods section.

n/a	Confirmed	
☐	☒	The exact sample size (*n*) for each experimental group/condition, given as a discrete number and unit of measurement
☐	☒	A statement on whether measurements were taken from distinct samples or whether the same sample was measured repeatedly
☐	☒	The statistical test(s) used AND whether they are one- or two-sided<br>*Only common tests should be described solely by name; describe more complex techniques in the Methods section.*
☐	☒	A description of all covariates tested
☒	☐	A description of any assumptions or corrections, such as tests of normality and adjustment for multiple comparisons
☒	☐	A full description of the statistical parameters including central tendency (e.g. means) or other basic estimates (e.g. regression coefficient) AND variation (e.g. standard deviation) or associated estimates of uncertainty (e.g. confidence intervals)
☐	☒	For null hypothesis testing, the test statistic (e.g. *F*, *t*, *r*) with confidence intervals, effect sizes, degrees of freedom and *P* value noted<br>*Give P values as exact values whenever suitable.*
☒	☐	For Bayesian analysis, information on the choice of priors and Markov chain Monte Carlo settings
☒	☐	For hierarchical and complex designs, identification of the appropriate level for tests and full reporting of outcomes
☒	☐	Estimates of effect sizes (e.g. Cohen's *d*, Pearson's *r*), indicating how they were calculated

Our web collection on statistics for biologists contains articles on many of the points above.

Software and code

Policy information about availability of computer code

Data collection	N/A
Data analysis	Intensity_Ratio_Nuclei_Cytoplasm.ijm2,3 was utilized for nuclear/cytoplasmic ratio analysis

For manuscripts utilizing custom algorithms or software that are central to the research but not yet described in published literature, software must be made available to editors and reviewers. We strongly encourage code deposition in a community repository (e.g. GitHub). See the Nature Research guidelines for submitting code & software for further information.

Data

Policy information about availability of data

All manuscripts must include a data availability statement. This statement should provide the following information, where applicable:

- Accession codes, unique identifiers, or web links for publicly available datasets
- A list of figures that have associated raw data
- A description of any restrictions on data availability

All data generated or analyzed during this study are included in this published article (and its supplementary information files) or available upon reasonable request.

Field-specific reporting

Life sciences study design

All studies must disclose on these points even when the disclosure is negative.

Sample size	Sample sizes were selected to ensure statistical significance and in agreement with published data. Sample sizes are shown in figure legends and as individual points on graphs.
Data exclusions	N/A
Replication	Each experiment was done in triplicate or more
Randomization	Controls were used when necessary
Blinding	N/A

Reporting for specific materials, systems and methods

We require information from authors about some types of materials, experimental systems and methods used in many studies. Here, indicate whether each material, system or method listed is relevant to your study. If you are not sure if a list item applies to your research, read the appropriate section before selecting a response.

Materials & experimental systems

n/a	Involved in the study	
☐	☒	Antibodies
☐	☒	Eukaryotic cell lines
☒	☐	Palaeontology and archaeology
☒	☐	Animals and other organisms
☒	☐	Human research participants
☒	☐	Clinical data
☒	☐	Dual use research of concern

Methods

n/a	Involved in the study	
☒	☐	ChIP-seq
☐	☒	Flow cytometry
☒	☐	MRI-based neuroimaging

Antibodies

Antibodies used	In depth details regarding every single antibody used in this study can be found in the methods section of the manuscript
Validation	The effectiveness of each antibody was tested via Knockdown experiments and western blotting analysis and have been used in published manuscripts.

Eukaryotic cell lines

Policy information about cell lines

Cell line source(s)	HEK293T, ATCC® CRL-3216™
Authentication	All cell lines were obtained from American Type Culture Collection (ATCC)
Mycoplasma contamination	All cell lines are tested for Mycoplasma contamination before use
Commonly misidentified lines (See ICLAC register)	N/A

Flow Cytometry

Plots

Confirm that:

- ☒ The axis labels state the marker and fluorochrome used (e.g. CD4-FITC).
- ☒ The axis scales are clearly visible. Include numbers along axes only for bottom left plot of group (a 'group' is an analysis of identical markers).
- ☒ All plots are contour plots with outliers or pseudocolor plots.
- ☒ A numerical value for number of cells or percentage (with statistics) is provided.

Methodology

Sample preparation	All samples were fixed in 70% ethanol at room temperature after first being washed twice with PBS.C ells were then stained with propidium iodide according to the Cold Spring Harbor Protocol (https://www.biolegend.com/en-us/protocols/propidium-iodide-cell-cycle-staining-protocol).
Instrument	LSRII-14 color and MofloAstrios
Software	FloJo
Cell population abundance	Cells deemed apoptotic/necrotic were exluded from analysis. mCherry expressing cells were deemed to be over 80% for mCherry based sorts and this number reaches 100% for propidium Iodide stained cells. >10,000 cells were used for all plots sho
Gating strategy	Negative (unstained cells) were utilized as negative controls to establish proper gating. All gating strategies were uniform for all included analysis. Doublet discrimination was performed by plotting FSC-H vs FSC-A and Propidium Iodide signal was utilized to sort for living healthy cells.

☒ Tick this box to confirm that a figure exemplifying the gating strategy is provided in the Supplementary Information.
